# What Is the Future for Global Case Management Guidelines for Common Childhood Diseases?

**DOI:** 10.1371/journal.pmed.0050241

**Published:** 2008-12-09

**Authors:** Mike English, J. Anthony G Scott

## Abstract

Mike English and J. Anthony G. Scott propose a framework for national surveillance, monitoring, and research that could help inform guideline development in low-income settings.

Others have made the point convincingly that Millennium Development Goal 4, a reinvigorated commitment to reduce child mortality by two-thirds before 2015, could be achieved if existing interventions were implemented on a massive scale [1,2]. Widespread access to simple therapeutic interventions, in accordance with World Heath Organization (WHO) case management guidelines, is a substantial part of this package. Assuming that the coverage of existing interventions can be improved, what will be the next major challenge? We believe it will be enabling groups of countries, individual countries, or even large states to take increasing responsibility for the future of their case management strategies. Here we bring together insights from a wide range of disciplines to propose a framework for national surveillance, monitoring, and research that could help inform guideline development in low-income settings. Although our focus is on childhood illness, the principles might be applied more widely.

## The History of Case Management Guidelines

WHO guidelines for common childhood illnesses were developed over several decades. A high burden of disease in settings with basic biomedical health systems demanded management strategies that were simple, safe, and inexpensive to achieve high coverage. Often the aim was high diagnostic sensitivity at the expense, to a degree, of specificity. However, much of the research underpinning the actual content of current guidelines was undertaken in the 1980s and early 1990s, and often considerable reliance was placed not on evidence but on expert opinion [3,4]. Three factors now undermine the relevance of this foundation.

### 1. Changing disease patterns.

In several developing countries, the incidence of malaria is falling sharply as health services achieve high coverage with insecticide-treated bed-nets and artemisinin-based combination therapies [5,6]. The introduction of the pneumococcal conjugate vaccine over the next decade, in addition to the conjugate vaccine against Haemophilus influenzae type b, will dramatically reduce the incidence of serious bacterial pneumonia in children [7–9]; meanwhile, the incidence of asthma is rising. Newer vaccines, urbanisation, infrastructure development, and poverty reduction strategies will all potentially further alter the epidemiology and relative burden of childhood diseases.

### 2. Health systems are heterogeneous and evolving differently.

Economic, social, and political developments are far from uniform across or even within low-income countries, and the capacities of their health systems vary commensurately. For example, the number of physicians per 100,000 population varies 10-fold even within Africa [10], and large variations exist in the scale and scope of the private health sector and the use of community health workers. Variability in health-seeking behaviour, access to care, types of service provider, and skill sets of health workers may all need to be taken into account in developing guidelines. For example, differentiating pneumonia from asthma may depend on a health worker's level of training.

### 3. New technologies demand new thinking.

Rapid technological innovation is producing an array of new diagnostics. While they have considerable potential to improve care [11], this potential may not always be realised. In Tanzania, introduction of a rapid, point-of-care malaria diagnostic did not reduce rates of malaria treatment for children with negative test results [12]. Thus, performance of new diagnostics may not be the same in the field as it is in carefully controlled clinical or laboratory tests for both technical and human reasons. We may seriously overestimate the effectiveness and cost-effectiveness of guidelines by failing to account for this discrepancy [13].

## The Science of Guideline Development

The science of guidelines is a relatively new discipline [14] that seeks to combine clinical, behavioural, and implementation research. New institutions such as the National Institute for Health and Clinical Excellence (http://www.nice.org.uk/) and the Agency for Healthcare Research and Quality (http://www.ahrq.gov/) have arisen to evaluate the population benefits of novel therapies. WHO has adopted this new science. In a 2006 journal supplement, WHO commissioned 16 reports to enunciate best practices for each step and methodology underpinning guideline development (http://www.health-policy-systems.com/articles/browse.asp?volume=4). We now illustrate some consequences of these changes in developing new guidelines for sick children.

### 1. Disease problems and therapeutic options.

The National Institute for Health and Clinical Excellence stresses the importance of a complete description of the problem tackled by guidelines [15]. For common childhood diseases in low-income countries, the relevant starting point is the child who develops a given symptom. For a population of such children, descriptive models can be developed to follow every possible course through access to care, diagnosis, treatment, and outcome and are elaborated as decision trees (see Figure 1 for a simple example). This form of population-level modelling has long been used in preventive interventions [16], in economic evaluation [17], and in clinical decision analysis [18]; recently, it has been used to evaluate the potential of new diagnostics [9,13]. However, its potential to answer questions about health systems as a whole has so far been largely ignored.

**Figure 1:**
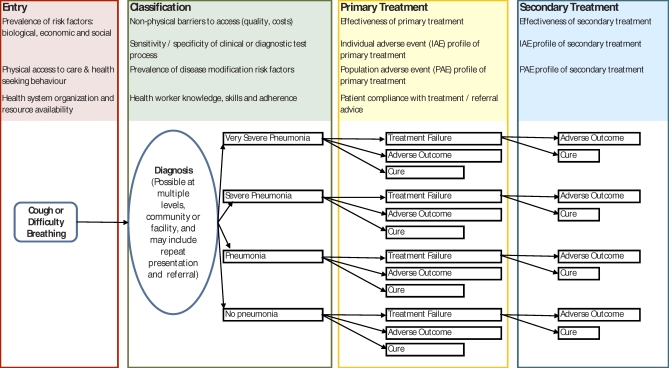
Simple Conceptual Framework for a Pneumonia Case Management Guideline, Based on Current WHO Advice, Illustrating Some of the Areas For Which Improved Global and Local Data Could Improve Understanding of Likely Policy Effectiveness Contextual factors are likely to operate from the stage at which they first appear on the left-hand side of the figure right through to the far right of the figure. The list shown is illustrative and not exhaustive. Consider the possible effects of introducing Haemophilus influenzae type b and pneumococcal conjugate vaccines. As the relative prevalence of bacterial pneumonia declines, the positive predictive value of any imperfect clinical or diagnostic test used to assign children to antibiotic treatment will also fall, and so the population benefits of antibiotic therapy will also decline. This will increase the proportion of unnecessary treatments and enhance the importance of adverse effects at both the individual and population levels. Population adverse effects, for example the development of resistance, are likely to reflect total antibiotic use and can be included as additional probabilities associated with each treatment episode. For clarity these are not shown in the illustration but see [11] for an example. The effect of our current lack of basic data may be illustrated when we try to estimate the value of new treatments in a new setting. In the last ten years, most emerging evidence has come from comparative efficacy trials of primary treatments for pneumonia. However, while we now have data on the relative reduction in risk of treatment failure or mortality attributable to a new therapy, we cannot estimate absolute risk reduction at a specific country level because data on the risk of treatment failure or death associated with the “old” treatment in this setting are missing. Consider also recent calls from paediatricians in areas with a high prevalence of HIV for changes in the guidelines on treating hospitalised infants with severe pneumonia. If we know baseline risks, the incremental effectiveness of any newly proposed treatment, and the prevalence of HIV, we can estimate the aggregate outcomes of changing treatment for a given population. Additionally, the net benefits of such a change could be compared with alternative interventions elsewhere in the decision tree, such as changing the antibiotic used for outpatient treatment of pneumonia.

Examining a complete model reveals how each step in a chain of events may be important and exposes unanticipated gaps in our evidence base. For example, a systematic review of the efficacy of oral therapy in pneumonia may provide robust evidence to guide one decision node, but effectiveness is dependent on the accuracy of health workers in classifying childhood pneumonia at this node. The right antibiotic given to the wrong patient does not constitute effective treatment.

The real power of these models, therefore, is in their ability to sum outcomes, favourable and unfavourable, for multiple scenarios represented by the proportion of the population of sick children moving down each of the possible linear paths. Building the model requires specification of the pathways to be considered and estimation of the probabilities that any fork in the path will be followed. By collecting appropriate information, we can therefore begin to understand how a guideline will affect health outcomes across an entire population. At nodes where data are scarce, sensitivity analyses can be conducted, varying the values incorporated at different points in the model within ranges based on available data or clinical plausibility. By varying the values used in models we can also help identify the key decision points and target research and resources at points that lead to the greatest overall benefit.

### 2. Constructing decision tree models.

The purpose of a model structure for guidelines is to facilitate decision making. In theory, guidelines reduce the latitude of clinical decisions by taking them out of the hands of health workers and placing them in the hands of a higher authority; but who should that authority be? Traditionally it has been WHO because guidelines have been created to apply to all low-income countries while tending to ignore their heterogeneity, a limitation WHO itself has already recognised. Actual policy decisions on guidelines are taken at country or even state level. These decisions are guided by: (a) The local incidence and aetiology of disease, and the prevalence of disease-modifying factors such as HIV; (b) National rates of uptake and coverage of new interventions such as bed-nets and vaccines; and (c) The organisation and capacity of a country's health system. To accommodate variation, therefore, decision models of childhood disease syndromes should incorporate the best available evidence and, where possible, reflect what is known about the local context. This does not mean that each county requires its own unique model. Generic approaches could be adapted for groups of countries, a single country, or even a single state in two major ways. Firstly, local data may be preferred in comparative assessments of alternative interventions and secondly, the models may be made more or less sophisticated reflecting both the complexity of the local situation and the availability of data. For example, models might incorporate treatment for Pneumocystis pneumonia based either on immediate HIV testing or on an empiric approach in cases of severe or very severe pneumonia.

This population modelling perspective is closely analogous to that of the policy maker who considers the net benefit to society when making decisions. Consequently, guidelines supported by decision tree models should be accessible to policy makers, especially if they incorporate local data on disease and context. Consider the example of a febrile child presenting to a health facility in Africa. What is the risk inherent in abandoning the policy of universal treatment with an anti-malarial drug when the true prevalence of malaria falls to, for example, one per hundred? Does the risk become acceptable if we introduce a rapid screening test with a sensitivity of 0.9? To what degree does minimising risks depend on good access to care and a health worker's capacity to arrange for review if symptoms persist or worsen (watchful waiting)? Such looming questions will be best answered by representative data on malaria prevalence and access to services and by local policy makers. There may be surprising results. For example, improving access to health care may turn out to be more effective and less expensive than introducing a new intervention. It is also possible to use these models to examine and compare the estimated effects of disease prevention strategies [19] that reduce the total number of cases entering the case management “pipeline.”

### 3. Incorporating data on cost.

Decisions on health care provision are always political and result in changes in cost to the provider, the patient, the population, or some combination of the three. Information on costs, including opportunity costs, is therefore a key element in decision making. Incorporating cost data also promotes transparency and protects against vested interests by illuminating the costs and consequences of alternative decisions. Unfortunately there are still very few local, basic cost data to incorporate into decision making, despite the efforts of WHO (http://www.who.int/choice/) and others. It should not be so difficult, from the provider perspective, to obtain appropriate data on, for example, the average “hotel” cost of a hospital day or the average cost of providing a primary care facility or a community health worker through which services are delivered. These types of data, when combined with service utilisation data (also often unavailable; see below), are essential to understanding the cost-effectiveness of programmes and should be quantified and made available by governments, donors, non-governmental organisations, and others.

### 4. Recognising the human factor.

Models of preventive interventions, such as vaccines, include parameters for vaccine effectiveness and vaccine coverage as primary data inputs. These data are gleaned from large-scale clinical trials and national surveys. Models of clinical guidelines also require data on the size of the intervention effect and the degree of coverage. However, they must also recognise that providers and recipients of health care do not or cannot always follow guidelines [12,20]. Research data on adherence and compliance in low-income settings are scarce [14,21], but health workers may be subject to a variety of external and personal interests that affect their practice. More generally, the “patient” is usually a somewhat abstract concept in the development of public health policies or guidelines. This makes it relatively acceptable to introduce guidelines that will fail a small proportion of all patients in the interests of doing the best for the large majority. Health workers, however, may reject such a guideline in the clinic because they are unwilling to pass on even a small risk to the population of patients they see. To counter this concern, models provide an effective means to communicate how rather abstract population benefits, such as delaying the development of antibiotic resistance, are affected by individual patient decisions like withholding antibiotic treatment. They also provide a means of assessing whether investing in adherence or compliance might be as effective as introducing new interventions. To understand the need for improved adherence or compliance in a setting demands some form of measurement. How many regions have any indicative data on whether their guidelines are actually followed?

### 5. Investing in local data.

Global burden of disease estimates are attracting much research attention. While initiatives such as the Health Metrics Network (http://www.who.int/healthmetrics/) aim to improve national health information systems, most countries are still unable to answer a simple question such as: “How many children with pneumonia are treated and die annually in hospital?” In a recent high-profile global modelling exercise, pneumonia case fatality rate estimates were based on only four published studies [11]. How can we quantify the absolute benefits of new interventions compared with “standard” treatment in a given country unless we know the baseline incidence of disease, risk of treatment failure, and case fatality rates? For example, consider a new antibiotic regimen for very severe pneumonia that is proven in large randomised controlled trials to reduce mortality risk by 25%. This relative benefit will result in very different absolute benefits where baseline case fatality rates for very severe pneumonia are 4% and 20%. In the former case, for every 100 children treated with a new, possibly expensive antibiotic one child death is averted, while in the latter case five are averted—dramatically influencing cost-effectiveness.

If we really wish to understand the potential impact of new interventions and policy decisions, there is an immediate need for basic community, clinical, and epidemiological data. Much of this data ought to be produced routinely by functional health systems. If data are unavailable or unreliable, then we should purposefully collect them at representative sentinel sites. In future, we should consider a handful of studies from across the globe inadequate to estimate important parameters such as the incidence of disease or the case fatality ratio. Data on disease burden can then be integrated with complementary data on costs, health systems, access to care, and coverage of interventions. The process of collecting and analysing these data should be co-ordinated increasingly by local public health institutions as part of parallel regional or national capacity building. This is essential if individual countries are to reap long-term benefits from better policy decisions.

## A Role for WHO

The development of WHO's first generation of simple case management guidelines represented the culmination of considerable formative research, and their impact has been considerable. However, no comprehensive process for evaluation, revision, or refinement was established at introduction, despite the fact that they are applied globally to hundreds of millions of episodes of pneumonia, diarrhoea, and malaria every year [22–24]. As the epidemiological landscape diversifies we will need appropriate evidence to adapt treatment guidelines for these millions of children so that the guidelines are optimally effective at regional or country, not continental, level. Strengthening this process may foster, through increased ownership, a virtuous cycle that generates demand for better data and improves the value and specification of models. As a starting point, models should be used to identify quickly those decision points and data that are most critical to the determination of costs and outcomes.

The transition to a more informed process of guideline development will take time. WHO is ideally positioned, however, to assist countries in an increasingly devolved process of decision making. It would be a retrograde step if each setting began to demand use of only its own data (and not what we are recommending). Systematic reviews of the evidence and strategically conducted research will be the most appropriate and efficient means to produce estimates for many model parameters. WHO should play an important role in providing such global evidence. We have argued that the value of guidelines will increasingly also depend on the quantity and quality of local data informing their development. Standardising and monitoring the collection and reporting of data coming from routine health system monitoring or targeted surveillance approaches would be of huge benefit, permitting data sharing across areas, and could be undertaken by WHO and its regional offices. Development of generic tools and analytical approaches allied to capacity building of local institutions would allow WHO and partners to foster progressively greater local responsibility for use of data and improvements in its scope and quality. WHO could share lessons learned and engage with international agencies to promote and provide unrestricted access to high-quality data.

## Conclusion

We have heard many calls for, and seen considerable resources devoted to, improving data to inform often vertical global health monitoring exercises. In order to determine health policy, industrialised countries are investing in systems to acquire their own data on the burden of diseases and the costs of interventions. This is not an expression of nationalism but a recognition that diseases and health systems vary across and even within countries. As low-income countries develop, they will also desire local data for this purpose to use in conjunction with other data available from international collaboration or synthesis. The question that we have posed is: how do we anticipate this need and its consequences for the evolution of health systems in low-income countries? We have argued that two simple approaches will carry this forward: the development of population-based models of health systems and the acquisition of relatively simple local data on the burden of disease, the effectiveness of simple curative practices, their cost, and pattern of usage. The models proposed combine international and local data and could lead, in turn, to a greater demand by policy makers for more and better data. Given the economic constraints it will be necessary to share data across regions for some time, especially the findings of major research studies. Initial investment in local health data will be required to foster the evolution of guidelines and policy in low-income countries. This challenges us to strengthen our support for clinical health systems research at country level, within ministries of health, and in public service and academic institutions. Low-income countries will need better trained epidemiologists, health economists, clinical epidemiologists, and behavioural and laboratory scientists who are able to gather, utilise, and interpret data from their region. The responsibility for this lies with all involved in improving global health and also depends on the countries themselves who, by application of simple health system models, create the demand for better care through better data.

## Abbreviations

WHO: World Heath Organization
